# Managing Respirable Quartz Exposure in Façade Renovations of Masonry Buildings

**DOI:** 10.3390/toxics14010018

**Published:** 2025-12-24

**Authors:** Tapani Tuomi, Kristiina Haapanen, Susanne K. Wiedmer

**Affiliations:** 1 Finnish Institute of Occupational Health (TYÖTERVEYSLAITOS), P.O. Box 40, FI-00032 Helsinki, Finland; 2 Department of Chemistry, University of Helsinki, P.O. Box 55, FI-00014 Helsinki, Finland

**Keywords:** respirable crystalline silica, construction work tasks, façade renovations, respirable quartz exposure, respirable dust exposure

## Abstract

Respirable quartz and dust exposures in dusty façade renovation work tasks were investigated. The presumption was that dust-producing work tasks can be performed safely, keeping exposures low, with practical, easily available methods to control dust emissions and exposure. The aim was to identify deficiencies in exposure management and compare exposure limiting methods to find out how to minimize dust emissions and exposures. Average respirable quartz and dust exposures from the 31 work situations, encompassing nine work tasks studied, were 0.082 and 1.3 mg/m^3^, respectively. Both values exceed the OEL in Finland, pointing to severe deficiencies in managing exposures. All tasks could, however, be executed safely, keeping exposures low. This often required using respirators while working inside façade covers or close to dust emissions. Other key things when planning exposure maintenance were the following: using water sprays and tool-specific exhausts vents; opening façade cover ventilation apertures; ensuring that non-participants in dusty work tasks are not exposed; working upwind from dust emissions; using pre-blended plaster; using grinders with extension handles; replacing diamond saws and angle grinders with hydraulic cutters when dismantling balcony elements; executing façade jackhammering with robots installed on lifting platforms prior to installing scaffolds and façade covers; detaching façade covers from the clean side; and using lifting platforms.

## 1. Introduction

According to the Finnish Job Exposure Matrix, ca. 43,700 workers were exposed to respirable quartz at the workplace in 2015, 84% of which were construction workers [1]. This constitutes about 36% of all construction workers in our country. Starting in 2020 the employers in Finland have a statutory obligation to yearly report those employees to the “Register of Persons Exposed to Substances and Methods that Cause Cancer at Work” (ASA register) who “have been exposed to a carcinogenic agent for a significant part of their working time on at least 20 working days” [2,3]. The employer must determine the exposure concentration either through occupational hygiene measurements or by assessing exposure based on surveys conducted under similar conditions [2,3]. In the absence of occupational hygiene measurement data, assessment of workers exposed to respirable crystalline silica in construction companies has been found to be challenging [4]. In 2022, a total of 22,296 people were reported as having been exposed to dust containing silica, of which 52% were employees in the construction sector [5]. The actual number is probably considerably higher, as indicated by the estimates above. Also, according to the Finland’s labor force survey, approximately 103,000 people worked as construction workers in Finland in 2022 [6]. In a previous study by the Finnish Institute of Occupational Health completed in 2022, 38% of the construction industry tasks studied (*n* = 148) exceeded the ASA registration threshold [4].

In occupational settings, the respirable fraction, i.e., the percentage of the inhalable fraction which is to be included at any given aerodynamic diameter, varies from 1.3% at 10 µm (0% at 16 µm) to 97% at 1 µm, with a so-called 50% cut-off at 4 µm [7]. Airborne respirable dust according to this convention is commonly extracted by using cyclones, with the air flow calibrated to correspond to a 50% cut-off at 4 µm [8].

The limit value for ASA registration of workers exposed to respirable crystalline silica has been established at 0.0050 mg/m^3^, which is 10% of the occupational exposure limit value (OEL) in Finland and 25% of the concentration known to be harmful [9,10]. In Finland, respirable silica concentrations in urban air are not studied in air quality studies. However, these measurements would not provide a directly comparable background concentration, since the 50% cut-off point of the collectors used in environmental sampling (PM2.5 and PM10) differs from the cut-off point of occupational respirable dust sample collectors. In the United States, the California State Office of Environmental Health Hazard Assessment (OEHHA) has set a limit value (0.0030 mg/m^3^) for silica concentration in urban air based on occupational epidemiological studies [11,12,13]. This limit value for crystalline silica concerns respirable crystalline silica, or in this case the PM4 fraction. As a result, researchers have adjusted the flow rate of the collectors used for environmental sampling and achieved the 50% cut-off point for 4 µm particles specified in ISO 7708 and EN 481 standards [7,14]. Richard and Bronzell have determined respirable crystalline silica concentrations in ambient air during a two-year measurement period in the vicinity of sand quarries using a Partisol 2000i collector (flow rate adjusted to 11.1 dm^3^/min) [15]. The annual average (avg.) silica concentrations measured from 24 h samples varied between 0.00022 and 0.00033 mg/m^3^ at the measurement sites and the highest measured concentrations over the two years were 0.00056–0.0021 mg/m^3^ [15,16].

Respirable crystalline silica concentrations in ambient air of urban environments have been studied since the 1980s [15,17]. However, more recent measurement data on respirable silica concentrations in urban air using modern sampling devices would be needed, as most recent studies appear to be based on PM10 data. The avg. silica concentrations calculated on the basis of individual measurement days in US cities varied from below the detection limit of 0.0019 mg/m^3^ for the PM2.5 fraction to 0.00090–0.0080 mg/m^3^ for the PM2.5-15 fraction [17]. The proportions of silica in the total mass of these particle fractions varied from 0 to 6% (PM2.5) to 0.30–23% (PM10) [17]. It is possible that quite high silica concentrations in the air would also be measured in Finnish cities during the street sanding season, as the highest measured particle concentrations in 2024 were 0.027 mg/m^3^ (PM2.5) and 0.16 mg/m^3^ (PM10) according to the air quality report of the Helsinki Region Environmental Services Joint Municipal Authority (HSY) [18]. However, it has been estimated that the ASA registration limit value in urban air would only be exceeded on a few days per year. Thus, concentrations above 0.0050 mg/m^3^ measured in personal samples are regarded as representing occupational exposure [19]. Based on risk of contracting silicosis [8], an avg. exposure concentration of 0.020 mg/m^3^, i.e., 40% of the OEL in Finland (0.050 mg/m^3^), is regarded as necessitating inclusion of workers to a yearly statutory health inspection, with testing of the respiratory capacity (spirometry) included [20].

According to the Finnish Occupational Disease Register, an avg. of seven cases of occupational silicosis are diagnosed each year, while an avg. of one lung cancer case related to respirable silica exposure is listed yearly [1]. However, not all cases of silicosis and lung cancers caused by silicosis are registered as occupational diseases, as insurance companies do not accept all diagnoses as occupational. The grounds for rejection have been the patient’s incomplete exposure history or insufficient evidence of an occupational causal relationship [1]. In the future, verifying workers’ occupational exposure may become easier, as more workers exposed to quartz and other respirable crystalline silicas are added to the ASA register.

Façade renovations of masonry buildings include many operations where exposure to quartz is potentially of particular concern. Such procedures include sand blasting, tuck pointing of mortar joints, replacement of façade joint fillers, jackhammering of plastered facades, jackhammering or cutting of façade blocks, removal of balcony elements, grinding of balcony floors, and three- and two-stage plastering. With very few exceptions, namely tuck pointing, jack-hammering, and concrete element/slab cutting, these include work tasks which have been sparsely studied [21]. The reason for these operations with accompanying work tasks raising concern is the scale of these tasks, in addition to the often high quartz content of materials removed or added, as well as the fact that many of the tasks are highly dust generating. Also, while these operations are performed outside, most of the time the façade is covered with a plastic sheeting or cover, inhibiting the dissipation of generated dust. Lastly, at least in Finland, use of water to manage dust exposure during façade renovations of masonry buildings is prohibited by most contractors due to mostly unsubstantiated fear of moisture damage.

The hypothesis in the present study was that even the dustiest of façade renovation work tasks can be performed safely, keeping exposures low, with practical exposure management solutions, without significantly increasing the budgets of building projects. Low in this context meant that quartz exposures are as a rule kept below the ASA registration limit (0.005 mg/m^3^), which is one tenth of the Finnish OEL (0.05 mg/m^3^) and one twentieth of the statutory exposure limit in Finland and other EU countries (0.1 mg/m^3^). At the very least, the exposure needs to be below the level of heightened risk of attaining silicosis and necessitating statutory health inspections, i.e., ca. 0.02 mg/m^3^, for career-long exposure [8]. The aim was to investigate exposure to respirable quartz and dust during façade renovations, particularly in work tasks that include highly dust-generating operations and methods, in order to find out how workers and contractors could manage dust emissions and minimize exposures in these work tasks. To this end, when called for based on the results, we compared different methods to carry out the same tasks and to use respiratory protection during dusty undertakings, as well as to protect from exposure via the general air. With the purpose of identifying deficiencies in exposure management and deducing how these tasks could be performed safely, keeping the exposure to respirable quartz and dust low. We did not strive to get an overview of exposure during façade renovation work tasks to be compared with other studies or to be used in risk assessment. We wanted to find out how, by using practical and easily applicable exposure control methods, dust-producing façade renovations could be performed without significant or excessive exposures that lead to a heightened risk of contracting silicosis or lung cancer over the course of a long working career.

## 2. Materials and Methods

### 2.1. Selecting Work Tasks and Sampling Sites

A total of 16 construction sites were visited on as many different days, mostly between 1 February and 31 August 2025 (Table 1). The sites were chosen in the larger Helsinki Metropolitan area (3698 km^2^, population 1.6 million), by contacting construction companies specialized in façade renovations. Ten active companies were contacted by phone. Out of these, three companies had suitable construction sites in the area and were willing to participate in the study. The work tasks selected for the study were those tasks in façade renovation of masonry buildings that were estimated by the companies contacted, as well as by the Confederation of Finnish Construction Industries RT (Helsinki, Finland), the Finnish Construction Trade Union (Helsinki, Finland), the Trade Union Pro (Helsinki, Finland), and the Finnish Occupational Safety and Health Administration (Hämeenlinna, Finland), to be those that are potentially associated with excessive exposure to respirable quartz. These trade unions represent the construction workers and foremen in Finland, the Confederation of Finnish Construction Industries represents the employers, while the Occupational Safety and Health Administration carries out inspections concerning the exposure of workers in Finland. These institutions also asked their expert members for comments on the practicability of the recommendations made based on the study.

The respirable quartz and dust exposure of workers during working days and during dust-generating tasks were measured separately by attaching two samplers to the breathing zone of each worker. A total of 31 workers (work situations) were involved in the study, working in nine different work tasks. We also measured the respirable quartz and dust concentrations in the general air within façade coverings and from lifting platforms, close to workers, from 15 stationary points. Stationary point samplers were placed in the vicinity of where dusty operations took place, usually on the same floor where the workers worked, at both ends of the scaffolds. In case the workers moved from floor to floor, stationary samplers were placed in the middle floor of the scaffolds, at both ends. In case there were no scaffolds or façade covers, general air samples were taken from the cages of lift trucks or vehicle cabins, where workers were exposed to respirable quartz through the general air. Three construction companies provided us with measuring sites in the Helsinki metropolitan area, including the cities of Helsinki, Espoo, and Kerava. The measurements were fairly evenly distributed amongst these companies: CONSTI Ltd. (Helsinki, Finland), MK Façade Ltd. (Vantaa, Finland), and Scanworks Ltd. (Vantaa, Finland). All measurements were performed without influencing dust management protocols or the worker’s use of respiratory protection, or other aspects potentially influencing exposure of worker to respirable dust or quartz.

### 2.2. Measuring Strategy

As most of the work tasks studied in the present investigation required respiratory protection during part of the workday, we attached two personal samplers to each worker. One of the samplers measured the avg. exposure during the whole workday, while the other was operated only during dust-producing tasks requiring the use of a respirator. As in a previous study [4], using the assigned protection factor of the respirator, we calculated the exposure of workers as follows (Equation (1)):

(1) $$E_{wd,2}={(C}_{wd}\times t_{wd}-C_{wp}\times t_{wp}\times {(PF}_{a}-1)/{PF}_{a})/t_{wp}$$

where the terms are defined as follows:

$C_{wd}$ is the avg. breathing zone concentration during the working day (mg/m^3^),
$C_{wp}$ is the avg. concentration during tasks requiring respiratory protection,
$t_{wd}$ is the sampling time of working day sample,
$t_{wp}$ is the sampling time of sample taken while using respiratory protection,
${PF}_{a}$ is the assigned protection factor of a respirator: 20 for FFP3 masks, 500 for face covering masks with P3 filters, 200 for P3 masks with assisted breathing, and 1000 for when air pressured combination masks were used,
$E_{wd,1}$ = $C_{wd}$ is the avg. working day exposure of workers who did not use a respirator,
$E_{wd,2}$ is the avg. exposure of workers during the working day, taking into account respiratory protection.
$E_{wd,3}$ = $E_{wd,2}$ = Estimate of exposure of workers working without respiratory protection if he or she had worn a respirator during dusty activities.

In addition, we calculated the percentage of the workers’ working day exposure concentrations ($C_{wd}$) of respirable quartz and dust, resulting from working without respirators during or outside of dusty processes (Equation (2)). If respirators were not used during dusty processes and to protect from high respirable quartz or dust concentrations in the general air, this percentage was 100%. If respirators were used throughout the working day, this percentage was 0%.

(2) $${\%\;of\;C}_{wd}={100\times (C}_{wd}\times t_{wd}-C_{wp}\times t_{wp})/{(C}_{wd}\times t_{wd})$$

### 2.3. Sampling and Analysis of Samples

Sampling of respirable dust was performed according to CEN and ISO standards [7,14]. The respirable quartz and dust exposure of workers during working days and during dust-generating tasks were measured with separate samplers, both attached to the breathing zone (upper chest) of workers, as described in EN 689 [22]. Briefly, respirable dust samples were collected with SKC GS-3 nylon cyclones (SKC Inc., Philadelphia, PA, USA) on 25 mm mixed cellulose ester membrane filters (Millipore AAWP025, 0.8 µm, Merck KGaA, Darmstadt, Germany). Flow rates of sampling pumps (SKC AirCheck) were calibrated to 2.75 dm^3^/min (±5%). Samples were taken from the breathing zone of workers and from stationary points at a height of approximately 1.5 m. The sampling time was at least 4 h, usually close to 8 h, to enable estimating the avg. exposure of workers during an 8 h working day. The hygienists were present during the measurements to monitor the use of respirators and to synchronize the sampling of the second sampler accordingly. As each worker already carried two sampling pumps and samplers, we were unable to perform replicate sampling.

Respirable dust was analyzed gravimetrically following ISO 15767 procedures [23]. Briefly, samples were conditioned in standard conditions (temperature 20 ± 2 °C, relative humidity 50 ± 5%) for a minimum of 24 h after first drying them in a desiccator for 2 days. Conditioned filters were weighed using a Mettler Toledo XPR36 precision balance (Mettler-Toledo AG, Greifensee, Switzerland) with a readability of 0.001 mg. The limit of quantitation (LOQ) for analyzing respirable dust was 0.060 mg/sample.

To remove calcite from samples prior to quartz analyses, 10 mL of 9% hydrochloric acid and 5 mL 2-propanol of analytical grade (VWR Chemicals, Paris, France) was added to samples in a Millipore ground joint flask for vacuum filtration (pore size 0.5 µm, diameter 25 mm) at room temperature, maintained at ca. 21 °C with a Technibel MAXFX246R51AA air-source heat pump (NIBE Industries Ltd., Markaryd, Sweden). Samples were left to stand for 3–5 min without stirring and filtered under suction, after which the filters were rinsed twice with 15 mL deionized water, transferred to a porcelain crucible, and allowed to dry overnight.

Crucibles containing the dried samples were covered with crucible covers and ashed (2 h, 600 °C). A total of 100 mg of oven-dried (110 °C, 24 h, stored in a desiccator) and mortar-ground potassium bromide (KBr) of infrared quality (Merck kGaA, Darmstadt, Germany) was added to the crucibles, before transferring samples to a mortar using a wooden spoon and a camel hair brush. The samples were ground using a pestle under a heat-generating lamp. Lastly, samples were transferred to a pellet-pressing platform, and the pellets were pressed manually using a Specac pellet press (Specac Ltd., Orpington, UK) with a tonnage load of 1.5 tons. This yielded pellets with a diameter of 5 mm and a thickness of 2 mm. Blank samples and control standards were prepared identically to samples. NIST SRM 1878b (The National Institute of Standards and Technology, Gaithersburg, MD, USA) were used to prepare standards.

Samples and calibrators were measured according to NIOSH method 7602 by measuring IR spectra in absorbance mode, using a Nicolet iS50 FTIR spectrometer (Thermo Fisher Scientific, Waltham, MA, USA) [24]. A control standard in the concentration interval of 2–100 µg was prepared with each sample batch from NIST SRM 1878 A standard powder added to KBr powder. The calibration curve was prepared from 1% and 2% (*w*/*w*) stock powders prepared from NIST SRM 1878 B standard powder added to KBr powder. From the stock solutions, 11 calibrators were prepared by adding amounts ranging from 2 µg to 100 µg of quartz in crucibles containing 25 mm mixed cellulose ester membrane filters. These calibrators containing the sampling filters were ashed at 600 °C and treated identically to workplace samples to yield an 11-point calibration curve spanning from 2 µg/sample to 100 µg/sample, with a Pearson correlation coefficient of 0.99 and a root mean square of calibration of 4.8 µg over the calibration range. The calibration was renewed if the result of the control standard diverged from the known amount by more than ±15% and after each annual service of the instrument. The pellet was scanned from 1000 cm^−1^ to 600 cm^−1^, and the peaks 775 and 800 cm^−1^ were used to verify the presence of quartz. Quantification was based on the mean peak height at 800 cm^−1^ of four consecutive measurements. Provided the four measurements from a pellet varied more than three times the standard variation from 60 random validation samples, the KBr tablet was reground, repressed, and remeasured. The LOQ of the quartz analysis was 2.0 µg/sample. Samples that exceeded the linear range of the analysis (100 µg/sample) were reground and diluted with KBr in sufficient amounts to reach the calibration range, by weighing 50 mg of the original sample in KBr powder and adding 50 mg of dried KBr, and carefully mixing the powder before repressing and remeasuring.

### 2.4. Exposure Classification

As to avg. working day exposures during a career (40 years), a four-to-five-stage risk classification of exposures explained in a previous paper [4] and adapted from BS18004 [25] was used in the present study (Table 2).

## 3. Results

### 3.1. Exposure Concentrations and Exposures

The mean respirable quartz exposure concentration for all work tasks was 0.21 mg/m^3^, while the median exposure concentration was 0.16 mg/m^3^ (Table 3). The mean was two times higher than the statutory limit value in the European Union and four times higher than the OEL in Finland, whereas the median exceeded the OEL by more than threefold. This underlines the need for either using respiratory protection in façade restorations or for adapting effective dust control methods to contain released respirable dust. The only work tasks where the respirable quartz exposure concentration did not exceed harmful levels were plastering and dismantling of façade covers. However, it should be noted that, on average, respirable dust exposure concentrations during plastering exceeded the OEL for respirable concrete dust (1 mg/m^3^) (Table 4). And this, in itself, necessitates effective measures to limit exposure during plastering. The highest exposure concentrations of respirable quartz (exceeding 0.1 mg/m^3^) were measured during sand blasting and dry ice blasting, joint seal removal, balcony floor grinding, and dismantling of balconies (Supplementary Table S1). Respectively, the highest respirable dust exposure concentrations (exceeding 1 mg/m^3^) were measured during sand blasting and dry ice blasting, joint seal removal, cutting of façade elements, balcony floor grinding, dismantling of balconies, and façade plastering (Table S1).

**Figure 1:**
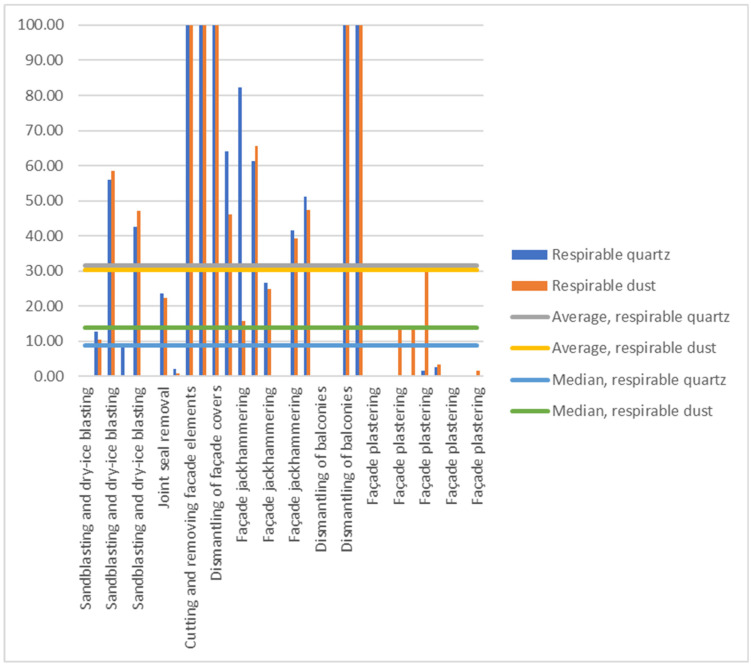
Percentage of the workers’ respirable quartz and dust working day concentrations, resulting from working without respirators during or outside of dusty processes.

On average, with respect to silicosis risk and/or exposure to respirable dust, exposure management was successful in dry ice blasting, joint seal removal, façade cover dismantling, and balcony floor grinding (Table 3 and Table 4). There were two main reasons leading to excessive exposures either to respirable quartz or dust. The first one being that respiratory protection was worn only during dust-releasing processes and not always when working inside façade coverings. Inside the façade covers, the average general air respirable quartz and dust concentrations were, on average, 0.49 mg/m^3^ and 4.2 mg/m^3^, respectively, with the respective median values (0.064 mg/m^3^ and 1.2 mg/m^3^) both exceeding the OEL. The general necessity to prolong the use of respirators is demonstrated also by comparing workday exposures to exposure concentrations (Table S1), as well by the percentage of respirable quartz and dust working day exposure concentrations, resulting from working without respirators (Figure 1). The other main reason for excessive respirable quartz or dust exposures was not using water or machine-specific exhaust vents during dust-producing operations. As to technical solutions, it seems clear that all of these work tasks should be performed before installing a façade cover, when possible. If not possible, all façade cover ventilation openings should be opened and, when feasible, workers should be situated up wind from dust sources (Table S1).

### 3.2. Work-Task-Specific Observations and Suggestions for Minimizing Exposure Management

**Figure 2:**
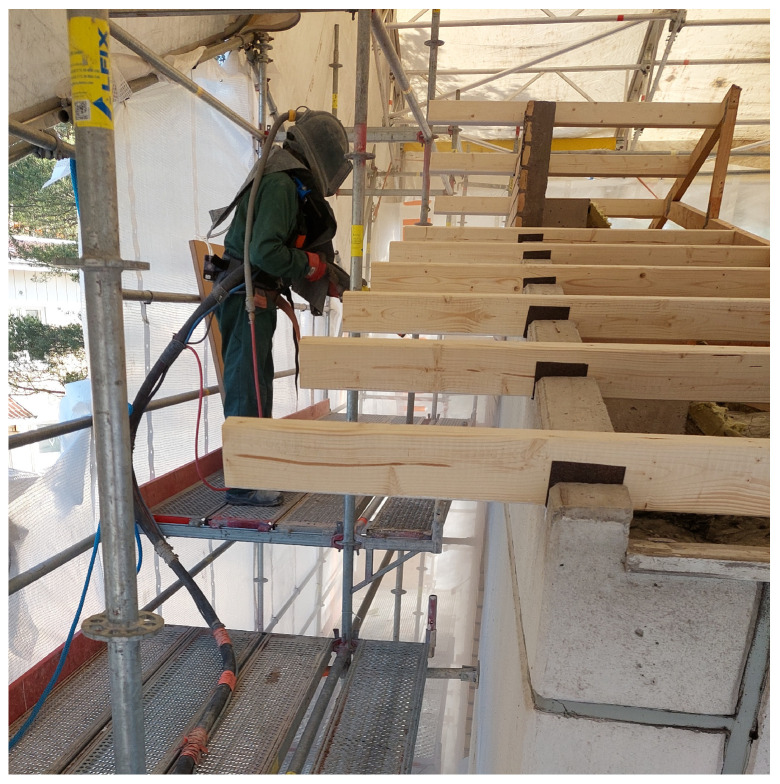
Wet sandblasting. The sandblaster needed to wear an air-fed sandblasting mask at all times inside the façade cover. Permission was obtained from the workers and their companies.

**Sandblasting**, as well as dry ice blasting were amongst the dustiest jobs encountered (Table 4, Figure 2). To minimize dust emissions, when sand is used instead of dry ice, wet sand is preferred. Regardless of the method of implementation and dust control measures, it was clear that sandblasting must be carried out using a fresh air mask or a blowing respirator with a P3 class particle filter (TH3 or TM3). Due to environmental protection regulations, sandblasting of facades must most often be carried out under a protective cover, in which case, in addition to the sandblaster, all those working under the protective cover, even for a short time, must wear a filtering half or full mask with at least a P3 class filter. In addition, the sandblaster and those assisting him must wear disposable protective overalls, protective gloves, and protective goggles. The assisting worker should wear the same protective equipment (Table S1). With these measures, both the respirable quartz and dust exposures of all workers involved can be reduced to safe levels, i.e., <0.0050 mg/m^3^ and <0.10 mg/m^3^, respectively.

**Figure 3:**
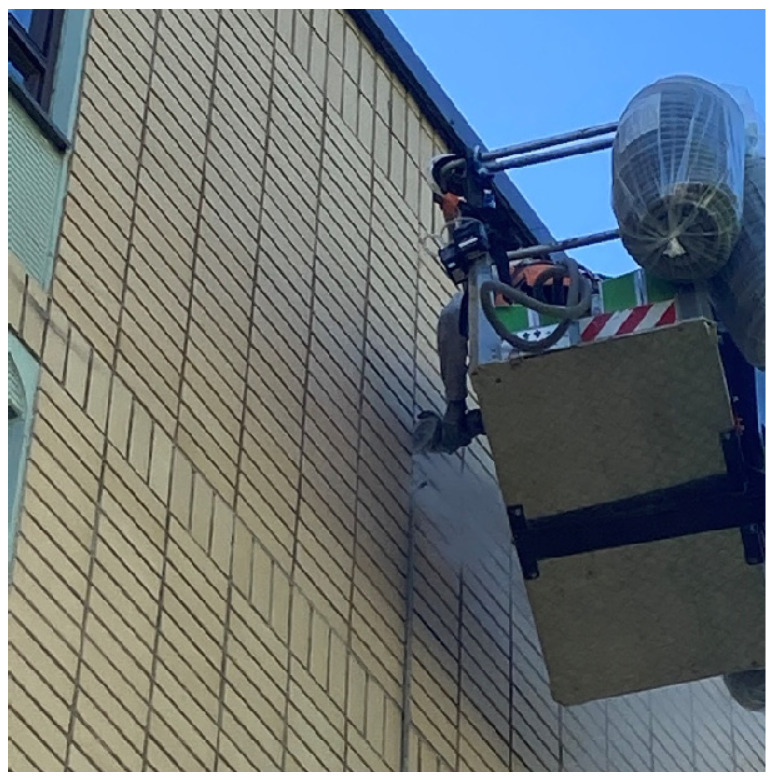
Joint seal removal. Angle grinding to clean the joint surfaces required using a respirator with P3-class filter. Permission was obtained from the workers and their companies.

When **removing joint seals**, the dustiest procedure was cleaning the adhesive surfaces of the expansion joint, particularly if it was performed with an angle grinder (Figure 3). Removing old joint sealants with a cutting blade or a drill can also generate some dust, but the most important thing in terms of dust control was to minimize the dust produced by the angle grinder and ensure the effectiveness of the respirator when using the grinder (Table S1). Consequently, the angle grinder should be equipped with a machine-specific exhaust vent. For respiratory protection, a filtering half or full mask with at least a P3-class filter is needed when cleaning the joint surfaces. When removing joints containing polychlorinated bifenyls (PCBs) and lead, in accordance with environmental regulations, the work must be performed under a protective cover, in which case, in addition to the worker removing the joints, all those working under the cover, even for a short time, need to wear the same protective equipment as the seal remover. Based on the general air samples, if a façade cover was not present, it was sufficient for only the worker removing the joint sealants to use a blowing respirator with a P3-class filter or a compressed air device, while others working in the vicinity can use a disposable FFP3 mask to contain quartz and dust exposures within safe limits (Table S1).

**Figure 4:**
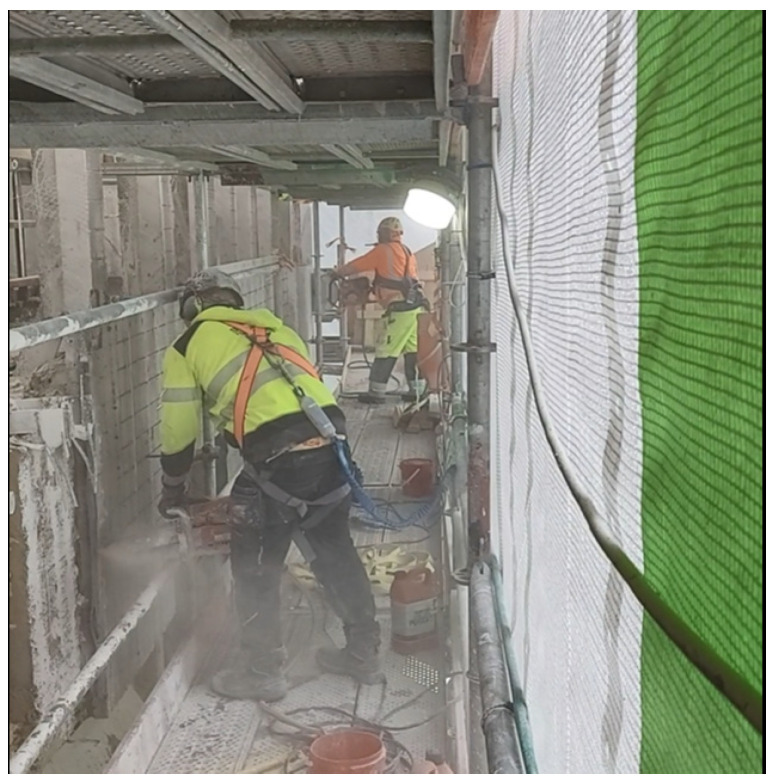
Wet cutting of facade elements produces an aerosol rich in respirable quartz and dust. As exhaust vents are only available for dry diamond saws, a respirator with a P3 class filter was a necessity. Permission was obtained from the workers and their companies.

**Diamond cutting and removing façade elements**, even when water is used to suppress dust production, was another particularly dusty undertaking (Table S1, Figure 4). Regardless of the method of execution and dust control measures, a fresh air mask or a blowing respirator with a P3 class particle filter (TH3 or TM3) must be used when performing these jobs. If the work is carried out under a façade cover, in addition to the diamond saw, all those working under the cover, even for a short time, must wear a filtering half or full mask with at least a P3 class filter (Table S1). Those who visit the cover for a short time must also wear at least a disposable FFP3 class respirator (half mask). With these precautions, both the respirable quartz and dust exposures of all workers involved can be reduced to safe levels.

**Figure 5:**
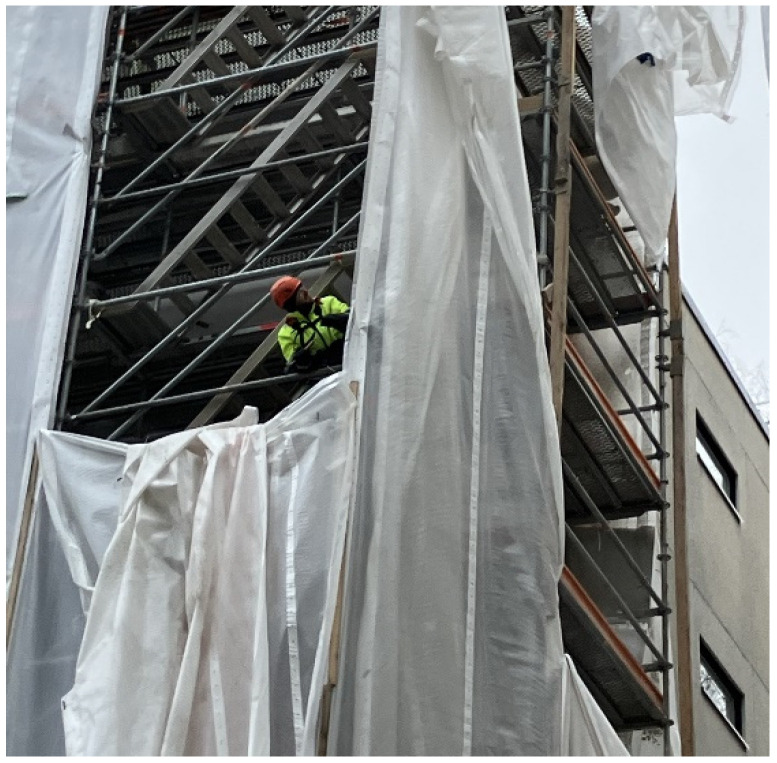
Dismantling of a façade cover. Cutting the cover and detaching mounting points inside the cover required using a FFP3 class half mask. Permission was obtained from the workers and their companies.

**Dismantling of façade covers** was not associated with particularly high exposures, but it was apparent that when working on scaffolds to detach a dirty façade cover from its mounting points inside the façade cover, a disposable FFP3 class respirator (half mask) is needed (Table S1, Figure 5). When possible, working from a lifting platform on the outside of the façade cover would be a less exposing solution in the dismantling of façade covers, due to the inside of the cover being dusty.

**Figure 6:**
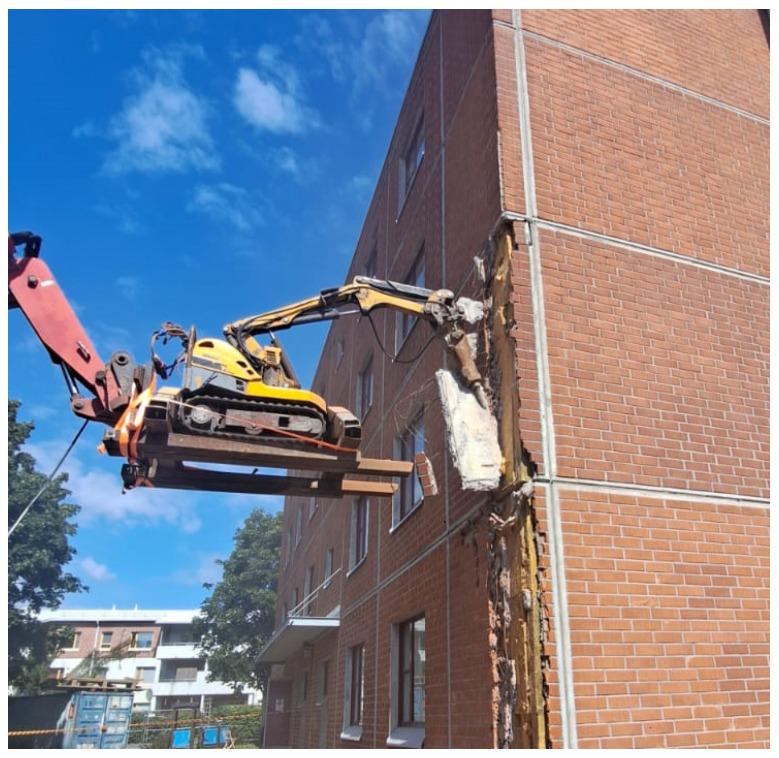
Façade jackhammering with a robot before installing scaffolds and façade covers ensured a safe distance between the operator and the dust source. Permission was obtained from the workers and their companies.

**Façade jackhammering** seems to often involve high or excessive exposures, particularly when performed inside a façade cover and provided that there are deficiencies in the use of respirators (Table S1). When using a manual jackhammer, a breathing mask with a P3 class filter (TH3P or TM3P) must be worn, regardless of whether spot chiseling is being performed or the entire facade is being chiseled (Table S1). Based on samples taken from the general air, we approximate that when working inside a façade cover, the breathing mask can be removed at the earliest about 30 min after the chiseling has ended, but this will depend on wind conditions inside the cover as well as on whether water spray is used or not (Table S1). Consequently, demolition workers working in the vicinity of the chiseler, inside the façade protection, must wear at least an FFP3 class respirator during chiseling and for about 30 min afterwards. Ventilation of the interior of the façade cover can be promoted, for example, by opening the ventilation hatches at the end of scaffolding floors or otherwise ensuring that there is throughflow in the space. The direction of the air flow should be considered so that chiseling dust is carried away from the worker. It is preferable to carry out jackhammering with a robot mounted on a lifting platform, without facade protection, before installing scaffoldings, and while observing sufficient safety zones (Figure 6). When jackhammering façade elements, robotic chiseling using a lifting device is also a safer method of implementation, as during the measurements, on two worksites by different companies, an element fell onto the scaffolds, creating a very dangerous situation for the workers. It should also be considered that traditional manual jackhammering is associated with intense vibratory stress on workers.

**Figure 7:**
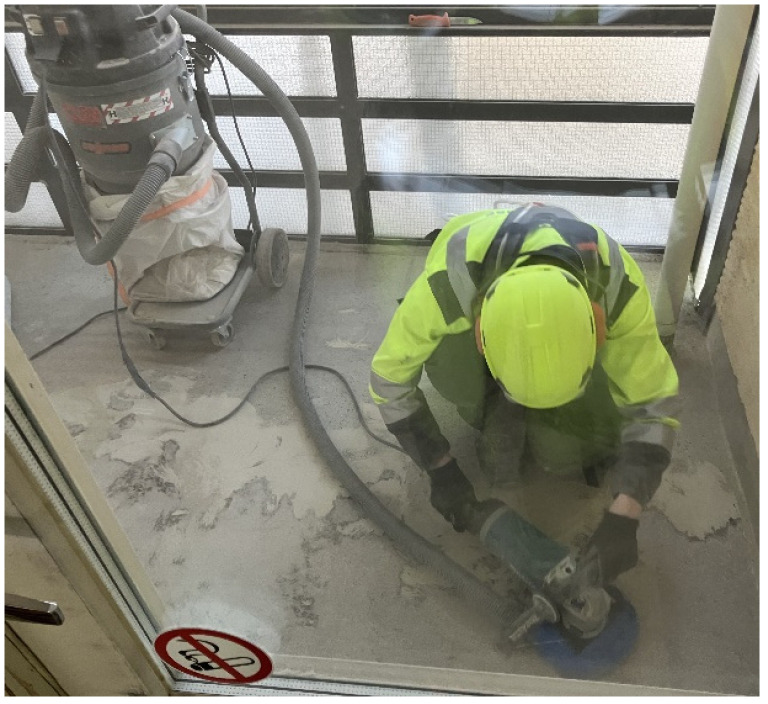
Grinding balcony floor corners with an angle grinder having a machine-specific exhaust vent. This required a respirator with a P3 class filter regardless of other control measures, as the dust source is close to the worker. Permission was obtained from the workers and their companies.

**Balcony floor grinding** was yet another task that required the use of respiratory protection regardless of extensive dust control measures (Table S1, Figure 7). Grinding was associated with high emissions of respirable dust despite dust control measures, wherefore the worker had to wear at least a half mask with a P3 filter during and after grinding. Grinding should mainly be carried out with a grinder with an extension handle, while a normal angle grinder should only be used in corners and other places, where a larger grinder cannot fit, or where it is necessary due to the surface structure. A machine-specific exhaust vent connected to a central dust extractor, whose collar or dust cover fits tightly to the machine, is needed to minimize emissions. If a half mask with at least a P3 filter was worn during grinding and for at least ½ hour after it, and the balcony is compartmentalized and underpressurized, we approximate that exposure will not rise above safe levels (Table S1).

**Figure 8:**
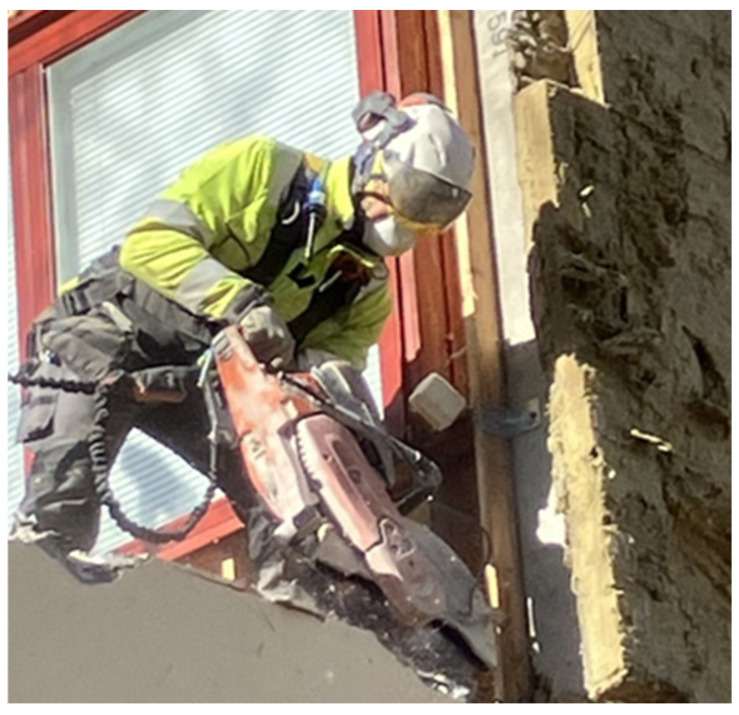
Diamond sawing to free balcony element mounting structures and cut reinforcing iron bars was the most dust-producing operation in the dismantling of balconies and should be replaced with hydraulic cutters, when possible. Permission was obtained from the workers and their companies.

**Dismantling of balconies** included many dust-generating tasks, including diamond sawing, drilling, angle grinding, and jackhammering. The work was by necessity performed without scaffoldings and façade covers being installed and hence, dust generated dissipated quickly even in fairly windless conditions. Nevertheless, it was clear that an FFP3-class respirator or a blowing respirator with a P3-class particle filter (TH3P or TM3P) should be used when performing these tasks (Table S1). Based on the general air samples, assisting workers must wear the same protective equipment if they are working on a lifting platform or near the respective dust source (Table S1). Further, it seemed apparent that instead of diamond cutting, it is advisable to use hydraulic cutters or other cutting methods that produce less dust and are associated with a lesser risk for accidents, taking into account the difficult working positions and the weight of a typical diamond saw (Figure 8).

**Figure 9:**
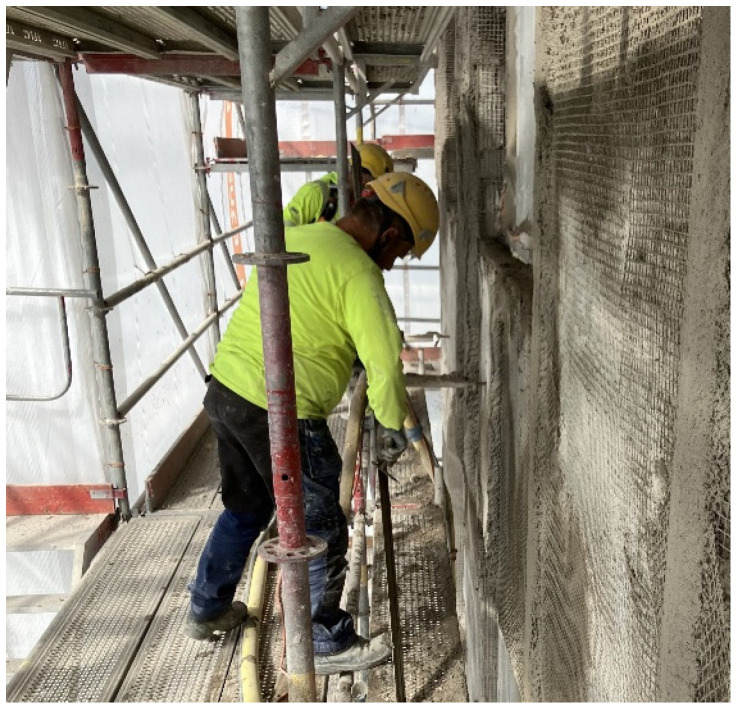
Spray plastering of a façade produces high concentrations of alkaline respirable dust inside the façade cover, requiring FFP3 class face masks to be worn, in addition to being mindful of the wind direction in relation to the dust source. Permission was obtained from the workers and their companies.

Lastly, with **façade plastering**, while generating high dust emissions during spraying of the plaster, and less so during finishing of sprayed surfaces, respirable quartz concentrations in the manually mixed plasters and pre-fabricated plasters used during the measurements seemed to be low (Table 3 and Table S1). Consequently, respirable quartz exposures were low to moderate, while respirable dust exposures were moderate to excessive (Table S1). Therefore, as a rule, both the spray plasterer and the workers finishing the sprayed plaster surface needed to wear disposable FFP3 respirators to protect from excessive exposure against the highly alkaline plasters (Table S1). And the same respirators should be used by all workers working on the scaffolds near the spray plasterer, since a façade cover needs to be installed during plastering (Figure 9). As with jackhammering, the workers should preferably be situated upwind from the dust source and throughflow should be enhanced by opening façade cover apertures.

## 4. Discussion

As has been reported previously in indoor construction, demolition, and infrastructure work tasks, a significant part of the respirable quartz and dust exposures during the façade renovations stemmed from either not wearing respiratory protection for long enough after dust-producing work tasks or to protect from exposure through high concentrations in the general air (Figure 1) [4,21,26]. It may be that breathing assisted respirators were not worn frequently enough and the basic filtering respirators most frequently used on the studied work sites (EN149, EN140 or EN 136, for instance); they also may not be as well-suited to physically demanding work tasks. Therefore, the workers preferred to work without them as much as possible. Also, it seemed clear that most of the time, the workers had not received adequate and effective training into the use of respirators, as described in the European quartz agreement practices [8]. In addition to respiratory protection, small changes to the way the tasks were scheduled, the recognition of wind direction with respect to the dust source, as well as the use of tool-integrated local exhaust ventilation and water, could have lowered the exposures significantly [27].

In a previous study, where we measured quartz and dust exposures in apartment building indoor work tasks, exterior work tasks, and infrastructure work tasks, the average respirable quartz exposure was 0.032 mg/m^3^, as opposed to 0.082 mg/m^3^ in the present study. Flanagan et al. measured respirable quartz exposures in the cutting of concrete bricks, joint grinding, jackhammering, and concrete mixing [26]. In these tasks, mean values were 0.080 mg/m^3^, 0.60 mg/m^3^, 0.15 mg/m^3^, and 0.040 mg/m^3^, respectively, with a total mean of 0.22 mg/m^3^ (median 0.11 mg/m^3^). In general, jackhammering of concrete outdoors has been reported to yield exposures from 0.0015 to 2.0 mg/m^3^, depending on the use of water, wind conditions, and whether exhaust vents are used or not [21]. In the present study we measured respirable quartz exposures in chiseling to be between 0.0050 mg/m^3^ and 0.081 mg/m^3^ (mean 0.022 mg/m^3^). Exposures in concrete slab cutting outdoors can range from 0.050 to 0.0050 mg/m^3^, again, depending on wind and the use of water and exhaust vents [21]. In the present study, cutting inside a façade cover exposed the cutter to an average concentration of 0.081 mg/m^3^ of respirable quartz, while the general air concentration was 0.064 mg/m^3^. Van Deursen et al. found the mean respirable quartz and dust exposures in tuck pointing to be 0.18 mg/m^3^ (range 0.020–0.80 mg/m^3^) and 3.4 mg/m^3^ (range 0.36–17 mg/m^3^), respectively [27]. For comparison, in the present study, exposures to respirable quartz and dust in joint cleaning were 0.0023–0.014 mg/m^3^ and 0.069–0.13 mg/m^3^, respectively. While we were not able to find comparative studies on the comprehensive renovation of masonry façades, with the aforementioned data and the variation within work tasks in the present study in mind, it is apparent that with the exception of façade cover dismantling, respirable quartz or dust exposures in all façade renovation tasks can range from low to excessive, based on the use of respiratory protection, as well as on all other measures taken to contain dust emitted and to minimize exposure.

When measuring respirable dust with accompanying quartz, the available sampling methods are associated with uncertainties. In the present study, we used SKC GS-3 cyclones, which have performed well in comparative studies [28]. The main problem encountered was that we had to make sure that the cyclone body with the airflow intakes did not bend when the workers leaned against railings or power tools. Also, as dust particles do not disseminate evenly in the vicinity of dust sources, the sampler closest to the dust source may possibly yield a higher concentration than a sampler hanging on the opposite side of the worker’s chest, further away from the dust-generating power tool [29]. Further, as the dust generated particularly in spray plastering and sand blasting was wet, the results for those work tasks may be underestimated [24,30]. To mitigate some of these uncertainties, samplers better suited for sampling respirable dust in work tasks with challenging body positions should be considered in similar studies as the present one. One example could be the SKC plastic cyclone with Higgins–Dewell design (225-69), which is identical in cut-off point and has a similar flow rate as the one used by us (SKC SG-3, 224-103). The Higgins–Dewell design is more compact and has a built-in space for the sampling filter, which should lower the risk for the cyclone becoming stuck and bent or the filter cassette being detached from the body of the cyclone through shear forces.

## 5. Conclusions

With very few exceptions, the work tasks studied required respiratory protection during part of the workday and in many cases throughout the whole working day. Particularly sandblasting, dry-ice blasting, cutting of façade elements, and balcony floor grinding were associated with high general air respirable quartz concentrations, in addition to high breathing zone concentrations. Furthermore, spray plastering of façades leads to high concentrations of alkaline respirable dust in general air as well as in the breathing zone of the spray plasterer and, on occasion, in the breathing zone of finishers as well. This underlines the importance of respiratory protection throughout the working day in these professions.

In other situations where exposures were excessive, the sources of the respirable quartz and/or dust exposures were close to the airways of the worker, while at the same time, there were insufficiencies in the selection and/or utilization of respirators. Typical examples of this were manual jackhammering, diamond cutting of façade elements, spray plastering, as well as cutting, drilling, grinding, and jackhammering during balcony dismantling.

Excluding balcony dismantling, performing the tasks without a façade cover would have, for the most part, resolved problems relating to high respirable quartz and dust concentrations in the general air. Particularly jackhammering of façades should preferably be performed prior to installing scaffolds and façade covers, by using a jackhammering robot installed on a lifting platform and operated from a safe distance. Joint seal removal is another example, where covering the façade may increase exposure. Some of the tasks, such as sandblasting, can only be performed inside a façade cover in many cities, due to environmental considerations, making respiratory protection the only means to contain exposure when using wet sand.

In the work tasks studied, the use of water and machine-specific exhaust vents could have been very helpful in lowering respirable quartz and dust exposures [4,21], but unfortunately, we were unable to verify their effectiveness due to fear of water damage by contractors and the lack of suitable tools by the operating companies. Jackhammer operators and workers using angle grinders, in particular, could have benefited from the use of exhaust vents and water sprays. Dry diamond cutting is another example of where exhaust vents would have lowered exposures of unprotected workers, but, particularly in balcony dismantling, challenging working conditions limited the use of vents and water with the heavy saws. In addition to the lack of water suppression in our sampling sites, the present study was limited by the relatively few measurements into each work task as well as the narrow geographical spread of the measurement sites. Therefore, this study cannot be seen to describe the general level or spread of exposures in these work tasks, but rather an attempt to demonstrate how the dust and quartz exposures could be managed in these tasks to minimize exposures. Albeit regarding the effectiveness of respirators to control exposures, the results in this study relied on calculations based on the protection factors of each respirator type, as reliable methods to measure quartz exposures inside the respirators are not available.

All in all, however, it seems that while many of the façade renovations studied can lead to significant or excessive exposures to either respirable quartz or dust, or both, they all could be executed safely, keeping both exposures low. This would have in many cases required always using respirators while working inside façade covers and/or close to dust emissions. Other key things to take into consideration when planning exposure maintenance protocols for these work task were in no particular order: (1) Using water sprays and tool-specific exhaust vents to contain dust emissions; (2)opening all façade cover ventilation openings; (3) planning work schedules in such a way that non-participants in dusty work tasks do not work inside the façade cover during such tasks or immediately after them; (4) making sure that workers are situated upwind from dust emissions whenever possible; (5) using pre-blended, ready to use plaster during plastering of façades; (6) Using grinders with extension handles when possible; (7) avoiding the use of diamond saws and angle grinders when dismantling balcony elements, and replacing them with hydraulic cutters if possible; (8) executing façade jackhammering with robots installed on lifting platforms prior to installing scaffolds and façade covers and replacing façade element cutting by diamond saws with automated jackhammering, as described above; and (9) when possible, detaching façade covers from the clean side, using lifting platforms.

To our understanding, most of these considerations can be taken into use without significant extra costs, and in the long run, all of them will cut costs, either by saving time and labor, such as point nr. 8, or by possibly cutting expenditures related to sick leave and health insurance.

## Figures and Tables

**Table 1:** Measurement sites.

	Operation		Nr. of Work Sites	Nr. of Work Tasks Involved
1	Sand blasting and dry-ice blasting		4	3
2	Removal of façade joint fillers		1	2
3	Dismantling of façade covers		1	2
4	Jackhammering of plastered facades		2	3
5	Jackhammering of façade elements		2	4
6	Cutting and removing façade elements		1	2
7	Removal of balcony elements		2	2
8	Three-stage plastering		3	3
9	Grinding of balcony floors		1	1
		Sum:	16	22

**Table 2:** (**a**) Classification of quartz exposure. (**b**) Classification of exposure to respirable dust [4].

Exposure (mg/m^3^)	% of OEL_8h_	Significance of Exposure
**a**		
<0.005	<10%	Low
0.005–0.02	10–40%	Moderate
0.02–0.05	40–100%	Significant
0.05–0.1	>100%	Excessive
>0.1	>200%	Exceeds statutory exposure limit
**b**		
<0.1	<10%	Low
0.1–0.5	10–50%	Moderate
0.5–1	40–100%	Significant
>1	>100%	Excessive

**Table 3:** Average respirable quartz concentrations and exposures in work tasks studied.

Work Task	Workday Exposure Concentrations on Avg. (mg/m^3^)	Workday Exposures on Avg. (mg/m^3^)	Concentrations in General Air on Avg. (mg/m^3^)
Sand blasting	0.73	0.28	1.8
Dry ice blasting ^1^	0.22	<0.0015	0.26
Joint seal removal ^2^	0.17	0.015	0.0019
Cutting and removing facade elements ^1^	0.081	0.081	0.064
Dismantling of façade covers	0.016	0.016	0.0032
Façade jackhammering	0.040	0.022	0.036
Balcony floor grinding ^1^	0.39	0.013	1.2
Dismantling of balconies ^2^	0.27	0.23	0.042
Façade plastering	0.0087	0.0033	0.0061 ^3^
All work tasks combined, on avg.	0.21	0.082	0.38
All work tasks combined, median	0.16	0.019	0.042
General air concentrations within façade coverings, on avg. (median)			0.49 (0.064)

^1^ Based on a single measurement; ^2^ No façade cover installed; ^3^ Approximated from the mean of the exposure concentrations of plaster finishers.

**Table 4:** Average respirable dust concentrations and exposures in work tasks studied.

Work Task	Workday Exposure Concentrations on Avg. (mg/m^3^)	Workday Exposures on Avg. (mg/m^3^)	Concentrations in General Air on Avg. (mg/m^3^)
Sand blasting	11	5.0	19
Dry ice blasting ^1^	8.1	<0.050	6.2
Joint seal removal ^2^	1.7	0.16	0.042
Cutting and removing facade elements ^1^	2.1	2.1	1.3
Dismantling of façade covers	0.27	0.27	0.040
Façade jackhammering	0.63	0.30	0.61
Balcony floor grinding ^1^	5.5	0.18	1.2
Dismantling of balconies ^2^	2.7	2.0	0.38
Façade plastering	1.3	0.69	0.74 ^3^
All work tasks combined, on avg.	3.7	1.3	3.3
All work tasks combined, median	2.1	0.50	0.74
General air concentrations within façade coverings, on avg. (median)			4.2 (1.2)

^1^ Based on a single measurement; ^2^ No façade cover installed; ^3^ Approximated from the mean of the exposure concentrations of plaster finishers.

## Data Availability

The data presented in this study are available on request from the corresponding author due to the data are not publicly available due to institute policy concerning information privacy.

## Supplementary Materials

The following supporting information can be downloaded at: https://www.mdpi.com/article/10.3390/toxics14010018/s1, Table S1: Respirable quartz and dust concentrations and exposures in different work tasks, conditions, and with respect to respiratory protection [4,31].

## Abbreviations

The following abbreviations are used in this manuscript:

AVG.	Average
OEL	Occupational Exposure Limit
