# Supplementary material for: Managing Respirable Quartz Exposure in Façade Renovations of Masonry Buildings
Source: Toxics. 2025 Dec 24;14(1):18. doi: 10.3390/toxics14010018 (PMC12845900; doi:10.3390/toxics14010018)
Supplement: Supplementary file 1 [file toxics-14-00018-s001.zip › toxics-4034168-supplementary.pdf]

Table S1. Respirable quartz and dust concentrations and exposures in different work tasks, conditions, and with respect to respiratory protection

			Respirable quartz (mg/m³)				Respirable dust (mg/m³)			
Title of worker or placement of general air sample	Work task and assignments	Conditions, dust management and personal protection	Exposure concentration during dusty processes	Avg. workday exposure concentration	Avg. workday exposure	Avg. workday exposure if respirator would have been worn[4]	Exposure concentration during dusty processes	Avg. workday exposure concentration	Avg. workday exposure	Avg. workday exposure if respirator would have been worn[4]
**Sandblasting and dry-ice blasting**										
Foreman	Observation and dust measurements during sandblasting. Outside of façade covering.	Covered façade with scaffolding and openings for ventilation.	-	0.0041	0.0041	nd	nd	0.11	0.11	nd
Sand blaster	Wet sandblasting of a painted facade to remove paint	Covered façade with scaffolding and ventilation openings. The sand was wetted. The worker used Tyvek protective overals, half-face mask with P3 class filter (EN140), sandblasting gogles and gloves.	0.0060	0.0055	0.00089	nd	0.19	0.17	0.023	nd
Sandblaster assistant	Assisting tasks related to wet sandblasting	Covered façade with scaffolding and openings for ventilation. The assistant worked mostly outside of the façade cover, but he did go inside the scaffolding on occasion. He did not wear a respirator.	0.12	0.21	0.21[1,2]	0.12	2.7	5.0	5.0[1,2]	2.9
Sand blaster	Wet sandblasting of the facade of a two-storied building to remove paint.	Covered façade with a deep scaffolding and openings for ventilation. The sand was wetted. The worker used a sandblasting hood with assisted breathing and Tyvek protective overals and gloves. Respiratory protection with assisted breathing was	0.97	0.79	0.073[1,2]	nd	15	9.3	0.073[1,2]	nd

		worn during sandblasting, but not at all times while inside the façade cover.							
Sand blaster	Wet sandblasting of the facade of a two-storied building to remove paint.	Covered façade with a deep scaffolding and openings for ventilation. The sand was wetted. A sandblasting hood with assisted breathing and Tyvek protective overalls and gloves were used. Respiratory protection was worn during sandblasting, but not comprehensibly while working inside the façade cover.	1.4	1.9	0.83[1,2]	nd	19	28	13[1,2]	nd
General air concentration inside the façade cover (1st floor) during sandblasting	Wet sandblasting of the facade of a two-storied building to remove paint.	Covered façade with a deep scaffolding and openings for ventilation. The sand was wetted (wet sandblasting).	-	2.7	2.7	nd	nd	45	45	nd
Concentrations in general air inside the façade cover (2nd floor) during sandblasting	Wet sandblasting of the facade of a two-storied building to remove paint.	Covered façade with a deep scaffolding and openings for ventilation. The sand was wetted (wet sandblasting).	-	1.4	1.4	nd	nd	19	19	nd
Concentrations in general air inside the faça-de cover while sandblasting	Wet sandblasting of a façade to remove paint.	Covered façade with a scaffolding and openings for ventilation. The sand was wetted (wet sandblasting).	-	1.4	1.4	nd	nd	19	19	nd

Dry-ice blaster	Dry-ice blasting of a façade to remove insulation left upon removal of façade elements and mineral wool boards	Covered façade with a scaffolding and vacuum units (ca. 1 000 m³/h) equipped with HEPA filters in the south end of each floor. A full-face mask with P3 class filter (EN136) was used for respiratory protection inside the façade covering.	0.22	0.22	<0.0015	nd	8.1	8.1	<0.050	nd
Concentrations in general air inside a façade cover during dry-ice blasting	Dry-ice blasting of a façade to remove insulation left upon removal of façade elements and mineral wool boards	Covered façade with a scaffolding and vacuum units (ca. 1 000 m³/h) equipped with HEPA filters in the south end of each floor.	nd	0.26	0.26	nd	nd	6.2	6.2	nd
**Joint seal removal**										
Joint sealer worker	Old masonry façade element joints were removed with a drill and the seam cleaned with an angle grinder prior to installation of new joint sealant.	The work was done from a lifting platform. No façade covering had been installed. A powered air purifying respirator with a TMP3 filter was used while using the angle grinder.	0.072	0.055	0.014	nd	0.72	0.54	0.13	nd
Joint sealer worker	Old masonry façade element joints were removed with a drill and the seam cleaned with an angle grinder prior to installation of new joint sealant.	The work was done from a lifting platform. No façade covering had been installed. A powered air purifying respirator with a TMP3 filter was used while using the angle grinder, as well as during all other dust-producing tasks.	0.31	0.11	<0.0023	nd	3.2	1.1	<0.069	nd
Concentrations in the general air during facade element joint removal.	Old masonry façade element joints were removed with a drill and the seam cleaned with an angle grinder prior to installation of new joint sealant.	The sample was installed at the back end of the lifting platform, from which old masonry façade element joint sealant was removed using a drill and the seam cleaned with an angle grinder.	nd	<0.0034	<0.0034	nd	nd	<0.10	<0.10	nd
Concentrations in the general air during	Old masonry façade element joints were removed with a drill and the seam cleaned with	The sample was installed at the back end of the lifting platform, from which old masonry façade	nd	0.0041	0.0041	nd	nd	<0.069	<0.069	nd

facade element joint removal.	an angle grinder prior to installation of new joint sealant.	element joint sealant was removed using a drill and the seam cleaned with an angle grinder.								
**Cutting and removing facade elements**										
Demolition worker	Wet sawing of facade elements with a diamond saw.	Covered façade with a scaffolding and vacuum units (ca. 1 000 m³/h) equipped with HEPA filters in the south end of each floor. The diamond saw was equipped with water feed. Respiratory protection was not used.	0.081	0.081	0.081[1,2]	<0.0017	nd	2.1	2.1[1,2]	<0.05
Concentrations in general air inside a façade cover during wet cutting of façade elements	Wet sawing of facade elements with a diamond saw.	Covered façade with a scaffolding and vacuum units (ca. 1 000 m³/h) equipped with HEPA filters in the south end of each floor. The diamond saw was equipped with water feed.	nd	0.064	0.064	nd	nd	1.3	1.3	nd
**Dismantling of façade covers**										
Carpenter	Separating façade coverings from their mounting points, including support planks, in addition to bringing them down and taking them to a demolition lorry.	The ventilation openings were for the most part closed. No respirators were used.	-	0.018	0.018	<0.0075	nd	<0.23	<0.23	<0.23
Carpenter	Separating façade coverings from their mounting points, including support planks, in addition to bringing them down and taking them to a demolition lorry.	The ventilation openings were for the most part closed. No respirators were used.	-	0.013	0.013	<0.0033	nd	0.43	0.43	<0.098
Concentrations in general air in the middle of the 1st floor	Separating façade coverings from their mounting points, including support planks, in addition to bringing them down and taking them to a demolition lorry.	The ventilation openings were closed, but air exchange improved considerably as the cover was brought down starting from the 8th floor.	nd	0.0035	0.0035	nd	nd	<0.080	<0.080	nd

during façade cover removal.									
Concentrations in general air at the south end of the 1st floor during façade cover removal.	Facade cover removal: separating façade coverings from their mounting points, including support planks, in addition to bringing them down and taking them to a demolition lorry.	The ventilation openings were closed, but air exchange improved considerably as the cover was brought down starting from the 8th floor.	nd	0.0028	0.0028	nd	nd	<0.080	<0.080	nd
**Façade jackhammering**									
Foreman	The day was spent mostly in the office, with occasional visits on the covered façade scaffoldings, where jackhammering was performed. One floor above the office, the plumbing was renewed in a restricted area inside an underpressurized enclosement.	The office was ventilated. Jackhammering was done without the use of water or machine-specific exhaust vents. A disposable FFP3-mask was used while inside the façade cover.	0.011	0.0077	0.0051	nd	0.30	0.14	0.068	nd
Jackhammerer	Local jackhammering of a plastered facade to remove plaster from damaged spots.	A hand-held jackhammer was used. It did not include an exhaust hose and water was not used to contain dust. A disposable FFP1-mask was used during jackhammering, but not at all times while inside the façade cover. The ventilation openings were closed.	0.0058	0.022	0.019	nd	0.84	0.67	0.25	nd
Jackhammerer	Jackhammering to remove brick-clad façade elements with a manual jackhammer.	The façade was covered. The ventilation openings were mostly closed. A half-face mask with a P3 class filter (EN140) was worn during jackhammering, but not at all times while working inside the façade cover. The jackhammer was not equipped with water feed or a machine- specific exhaust.	0.076	0.13	0.081	nd	0.78	1.5	1.0	nd

Jackhammerer	Jackhammering to remove brick-clad facade elements with a BROKK 60 demolition robot.	The façade was covered. The ventilation openings were mostly closed. A half-face mask with a P3 class filter (EN140) was worn during jackhammering, but not at all times inside the façade cover. The demolition robot was operated with a remote control above the wind, in relation to the robot. The jackhammer was not equipped with water feed or a machine specific exhaust.	0.041	0.037	0.011	nd	0.68	0.60	0.16	nd
Jackhammerer	Jackhammering to remove brick-clad facade elements with a BROKK 60 demolition robot mounted on a platform prior to installing scaffoldings and façade covers.	No façade covering, no scaffolding. The robot was operated from the ground, within a ventilated vehicle cabin.	<0.0050[3]	<0.0050[3]	<0.0050[3]	nd	<0.10[3]	<0.10[3]	<0.10[3]	nd
Demolition worker	Jackhammering to remove brick-clad façade elements with a manual jackhammer in addition to removal of demolition waste and other assisting tasks.	The façade was covered. The ventilation openings were mostly closed. A half-face mask with a P3 class filter (EN140) was worn during jackhammering, but not at all times inside the façade cover. The jackhammer was not equipped with water feed or a machine specific exhaust.	0.025	0.038	0.015	nd	0.54	0.79	0.30	nd
Concentrations in the general air during local, manual jackhammerring of a plastered façade.		The façade was covered and ventilation openings were mostly closed. A hand-held jackhammer was used. It did not include an exhaust hose and water was not used to contain dust. The ventilation openings were closed.	nd	0.033	0.033	nd	nd	0.49	0.49	nd
Concentrations in the general air during	Jackhammering to remove brick-clad façade elements with a manual jackhammer and a robot.	The façade was covered. The ventilation openings were mostly closed. The jackhammers used were not equipped with water feed or machine-specific	nd	0.039	0.039	nd	nd	0.72	0.72	nd

façade jackhammering .		exhausts. The sampler was situated above the wind, in relation to the jackhammers.								
**Balcony floor grinding**										
Carpenter	Grinding of balcony floors with an angle grinder and local jackhammering of a plastered facade.	An H-class industrial vacuum cleaner was connected to the grinder and a vacuum unit (800 m³/h) was used to underpressurize the enclosed balcony. A half-face mask with P3 class filter (EN140) was worn during grinding and while on the balcony.	0.25	0.39	0.013	0.21	3.80	5.5	0.18	nd
Concentrations in the general air of an enclosed and underpressurized balcony during floor grinding.	Grinding of balcony floors with an angle grinder	An H-class industrial vacuum cleaner was connected to the grinder and a vacuum unit (800 m³/h) was used to underpressurize the enclosed balcony.	nd	0.019	0.019	nd	nd	1.2	1.2	nd
**Dismantling of balconies**										
Foreman/demolition worker	Dismantling of balcony elements. Holes for lifting cables were drilled into the floor elements. The cast-in brackets were exposed with a jackhammer and cut with a diamond saw. Reinforcements were cut with an angle grinder. Elements were detached using a hydraulic spreader and lifted to the ground with a crane. Angle irons were attached to window frames by drilling after removing the	The tools used did not have device-specific exhaust ventilation. Cutting, drilling and chiseling were done dry. FFP2 disposable masks were used in the work. As there were no façade cover, dust produced dissipated quickly.	0.13	0.13	0.013	nd	2.0	2.0	0.20	nd

	balcony elements. The insulation was removed from around the balcony.									
Demolition worker	Dismantling of balcony elements. Holes for lifting cables were drilled into the floor elements. The cast-in brackets were exposed with a jackhammer and cut with a diamond saw. Reinforcements were cut with an angle grinder and elements were detached using a hydraulic spreader and lifted to the ground with a crane. Angle irons were attached to window frames by drilling after balcony elements were removed. The insulation was removed from around the balcony.	The tools used did not have device-specific exhaust ventilation. Cutting, drilling and chiseling were done dry. FFP2 disposable masks were used in the work. As there were no façade cover, dust produced dissipated quickly.	0.19	0.19	0.019	nd	1.4	1.4	0.14	nd
Demolition worker	Dismantling of balcony elements. Holes for lifting cables had been pre-drilled into floor elements. The cast-in brackets were exposed with a jackhammer and cut with a diamond saw. Reinforcements were cut with an angle grinder. The elements were detached using a hydraulic spreader and lifted to the ground with a crane. Angle irons were attached to window frames by drilling after balcony elements were removed. Insulation was removed from around the balcony.	Respirators were not worn, cutting, drilling and jackhammering were performed without the use of water or machine-spesific exhaust vents. The façade was not covered, and the weather was sunny and conditions windstill (19° C, 2-3 m/s).	0.87	0.46	0.57		7.2	5.0	5.3	0.25
Demolition worker	Dismantling of balcony elements. Holes for lifting cables had been pre-drilled. The cast-in brackets were exposed with a jackhammer and cut with a diamond saw. Reinforcements were	Respirators were not worn, cutting, drilling and jackhammering were performed without the use of water or machine-spesific exhaust vents. The façade	0.38	0.30	0.30	0.015	3.4	2.5	2.5	0.13

	cut with an angle grinder. Elements were detached using a hydraulic spreader and lifted to the ground with a crane. Angle irons were attached to window frames by drilling after the balcony elements were removed. Insulation was removed from around the balcony. During the morning, four balconies were removed, while the afternoon was spent dismantling roof elements from balconies after first removing the bitumen membranes covering them.	was not covered, and the weather was sunny and conditions windstill (19° C, 2-3 m/s).								
Concentrations in the general air during dismantling of balcony elements.	Dismantling of balcony elements. Holes for attaching lifting cables had been pre-drilled into the floor elements. The cast-in brackets were exposed with a jackhammer and cut with a diamond saw. The reinforcements were cut with an angle grinder. Elements were detached using a hydraulic spreader and lifted to the ground with a crane. Angle irons were attached to the window frames by drilling after the balcony elements were removed. The insulation was removed from around the balcony.	The sample was installed at the back end of the lifting platform, from which demolition workers entered balconies and did some of the operations involved. Cutting, drilling and jackhammering were performed without the use of water or machine-specific exhaust vents. The façade was not covered, and the weather was sunny and conditions windstill (19° C, 2-3 m/s).	nd	0.060	0.060	nd	nd	0.53	0.53	nd
Concentrations in the general air during dismantling of balcony elements.	Dismantling of balcony elements. Holes for attaching lifting cables had been pre-drilled into the floor elements. The cast-in brackets were exposed with a jackhammer and cut with a diamond saw. The reinforcements were cut with an angle grinder. Elements were detached using a hydraulic spreader and lifted to the ground with a crane. Angle irons were attached to the window frames by drilling after the balcony elements were removed. The insulation was removed from around the balcony.	The sample was installed at the back end of the lifting platform, from which demolition workers entered balconies and did some of the operations involved. Cutting, drilling and jackhammering were performed without the use of water or machine-specific exhaust vents. The façade was not	nd	0.023	0.023	nd	nd	0.22	0.22	nd

	ground with a crane. Angle irons were attached to the window frames by drilling after the balcony elements were removed. The insulation was removed from around the balcony.	covered, and the weather was sunny and conditions windstill (19° C, 2-3 m/s).								
		**Façade plastering**								
Plasterer	Showeling wet mortar and dry cement into a concrete mixer at a mixing ratio of 9:1 and feeding into a trough.	Use of wet concrete and a FFP3 respirator when working at the mixing point. The mixing point was situated outside, under a rain cover, allowing for free air flow from all sides.	<0.0016	<0.0016	<0.0016	nd	0.67	0.40	0.027	nd
Plasterer	Finishing sprayed plaster surfaces inside a façade cover with a finishing trowel, corner trowel and a window trowel. Working next to a spray plasterer.	No control measures or respiratory protection. The façade cover ventilation openings were for the most part closed.	<0.0016	<0.0016	<0.0016	<0.0016	0.49	0.49	0.49	0.024
Plasterer	Spray plastering (floating coat, 2nd stage of three-layer plastering). Spraying plaster mortar onto the façade with the help of compressed air.	No control measures or respiratory protection. The façade cover ventilation openings were for the most part closed.	0.014	0.012	0.012	<0.0016	3.1	3.1	3.1	0.16
Plasterer	Shoveling wet mortar and dry cement into a concrete mixer at a mixing ratio of 6:1 and feeding into a trough.	Use of wet concrete. The mixing point was situated outside, under a rain cover, allowing for free air flow from one side. A respirator was not in use.	<0.0018	<0.0015	<0.0015	<0.0015	0.44	0.43	0.43	0.076
Plasterer	Finishing sprayed plaster surfaces inside a façade cover with a finishing trowel, sponge float, corner trowel and a window trowel. Working next to a spray plasterer.	No control measures or respiratory protection. The façade cover ventilation openings were for the most part closed.	<0.0017	<0.0015	<0.0015	<0.0015	0.58	0.74	0.74	0.26
Plasterer	Spray plastering (finishing coat, 3rd stage of three-layer plastering). Spraying plaster mortar onto the façade with the help of compressed air.	No control measures or respiratory protection. The façade cover ventilation openings were for the most part closed.	0.014	0.012	0.012	<0.0015	1.03	0.89	0.89	0.045

Plasterer	Finishing sprayed plaster surfaces inside a façade cover with a finishing trowel, sponge float, corner trowel and a window trowel. Working next to a spray plasterer.	The plaster was delivered ready to use in a bin. All façade cover ventilation openings were opened. A FFP2 respirator was worn during plastering.	0.020	0.020	0.0010	nd	0.88	0.88	0.088	nd
Plasterer	Finishing sprayed plaster surfaces inside a façade cover with a finishing trowel, sponge float, corner trowel and a window trowel. Working next to a spray plasterer.	The plaster was delivered ready to use in a bin. All façade cover ventilation openings were opened. A FFP2 respirator was worn during plastering.	0.0029	0.0029	0.00029	nd	0.86	0.86	0.086	nd
Plasterer	Spray plastering (floating coat, 2nd stage of three-layer plastering). Spraying plaster mortar onto the façade with the help of compressed air.	The plaster was delivered ready to use in a bin. All façade cover ventilation openings were opened. A FFP2 respirator was worn during plastering.	0.028	0.028	0.0014	nd	3.6	3.6	0.36	nd
Concentrations in general air during spray plastering.	Spray plastering of a façade.	The façade was covered, some of the ventilation openings were opened.	nd	0.0061	0.0061	nd	nd	0.74	0.74	nd

[1]Measurements from 2021-2022; [2]These four exposure data were included in one of our previous publications [4] and reprinted with permission; [3]The cabin windows were closed and the cabin air filtered similarly to what is described in SFS-EN 15695-1 [31]; [4]This could not be calculated, in case respirators were used (marked as "nd").
